# Identification of candidate genes and enriched biological functions for feed efficiency traits by integrating plasma metabolites and imputed whole genome sequence variants in beef cattle

**DOI:** 10.1186/s12864-021-08064-5

**Published:** 2021-11-15

**Authors:** Jiyuan Li, Robert Mukiibi, Yining Wang, Graham S. Plastow, Changxi Li

**Affiliations:** 1 Department of Agriculture, Food & Nutritional Science, University of Alberta, T6G 2P5 Edmonton, Alberta Canada; 2 The Roslin Institute and Royal (Dick) School of Veterinary Studies, University of Edinburgh, Edinburgh, Scotland, UK; 3 Lacombe Research and Development Centre, Agriculture and Agri-Food Canada, 6000 C&E Trail, Alberta T4L 1W1 Lacombe, Canada

**Keywords:** Feed efficiency, Metabolites, Metabolome-genome wide association studies, Candidate genes, Biological function enrichment analyses

## Abstract

**Background:**

Feed efficiency is one of the key determinants of beef industry profitability and sustainability. However, the cellular and molecular background behind feed efficiency is largely unknown. This study combines imputed whole genome DNA variants and 31 plasma metabolites to dissect genes and biological functions/processes that are associated with residual feed intake (RFI) and its component traits including daily dry matter intake (DMI), average daily gain (ADG), and metabolic body weight (MWT) in beef cattle.

**Results:**

Regression analyses between feed efficiency traits and plasma metabolites in a population of 493 crossbred beef cattle identified 5 (L-valine, lysine, L-tyrosine, L-isoleucine, and L-leucine), 4 (lysine, L-lactic acid, L-tyrosine, and choline), 1 (citric acid), and 4 (L-glutamine, glycine, citric acid, and dimethyl sulfone) plasma metabolites associated with RFI, DMI, ADG, and MWT (*P*-value < 0.1), respectively. Combining the results of metabolome-genome wide association studies using 10,488,742 imputed SNPs, 40, 66, 15, and 40 unique candidate genes were identified as associated with RFI, DMI, ADG, and MWT (*P*-value < 1 × 10^−5^), respectively. These candidate genes were found to be involved in some key metabolic processes including metabolism of lipids, molecular transportation, cellular function and maintenance, cell morphology and biochemistry of small molecules.

**Conclusions:**

This study identified metabolites, candidate genes and enriched biological functions/processes associated with RFI and its component traits through the integrative analyses of metabolites with phenotypic traits and DNA variants. Our findings could enhance the understanding of biochemical mechanisms of feed efficiency traits and could lead to improvement of genomic prediction accuracy via incorporating metabolite data.

**Supplementary Information:**

The online version contains supplementary material available at 10.1186/s12864-021-08064-5.

## Background

Feeding-related costs are the major expense in beef cattle enterprises, representing 55 % - 75 % of total production costs [1–3]. Reducing feed inputs per unit of production could significantly improve profitability by 9 to 33 % in beef production [4]. Additionally, with the projected increase of the global population to 9.6 billion by the year 2050, the growing demand for beef is likely to put more pressure on already limited production resources such as water, land, fertilizers and labor [5]. Moreover, studies have shown that more feed efficient beef cattle consume less feed for the same amount of beef produced, and meanwhile, have a reduced methane emission [6]. Therefore, improvements in feed efficiency of beef cattle can increase producer profitability and simultaneously lower the environmental footprint of beef production.

Residual feed intake (RFI) is an important indicator of feed efficiency, which is usually defined as the difference between an animal’s actual daily dry matter intake (DMI) and the expected daily DMI given the animal’s average daily gain (ADG) and metabolic body weight (MWT) [7]. Currently, measuring individual animal feed intake to calculate RFI is a complex and expensive process. Numerous studies in beef cattle have revealed moderate to high heritability estimates (0.16-0.68) for RFI [8–11], and thus make RFI suitable for genetic/genomic selection of efficient beef cattle. Over the decades, genome-wide association studies (GWAS) have detected thousands of single nucleotide polymorphisms (SNPs) and hundreds of candidate genes associated with RFI in beef cattle [12–15]. However, cellular and molecular functions associated with transcriptomic, metabolomic and proteomic levels of omic data, and detailed knowledge regarding the biological processes involved in feed efficiency still remain largely unknown. Metabolites are substrates or products of metabolic processes and are the results of combined endogenous and exogenous production [16], thus metabolites are considered as intermediate phenotypes between the genomic (base) and phenotypic (top) levels [16]. Integration of metabolomic data into feed efficiency studies could help reveal the relationship between animal genetics and physiological phenotypes (i.e. RFI and its component traits), thereby increasing the fundamental understanding of biological functions related to feed efficiency and improving genetic/genomic selection efficacy in beef cattle. Therefore, the objective of this study was to use metabolites as intermediate phenotypes to study genes and biological functions/processes related to feed efficiency in beef cattle. In this study, feed efficiency data were collected from a beef cattle population consisting of 493 crossbred bulls, heifers, and steers. Thirty-one metabolites and their concentration levels (µM) were quantified from plasma of these animals on the first day of feedlot tests. Linear regression models were applied to identify metabolites associated with RFI and its component traits (DMI, ADG, and MWT). Whole genome sequence variants were imputed and used in metabolome-genome wide association studies (mGWAS) to identify significant SNPs for trait associated metabolites. Candidate genes were mapped based on significant SNPs and gene functional enrichment analyses were subsequently performed on candidate genes of each trait to predict biological functions/processes associated with feed efficiency in beef cattle.

## Results

### Associations between feed efficiency traits and metabolites

Of the 31 metabolites analyzed, 11 were found to be significantly associated with the feed efficiency traits (*P*-value < 0.1) and the results of regression analyses are shown in Table 1. Among the significantly associated metabolites with each trait, ten metabolites showed *P*-values less than 0.05, and four metabolites (choline for DMI, glycine, citric acid, and dimethyl sulfone for MWT) showed *P*-values ranging from 0.05 to 0.1 (0.09, 0.05, 0.06, and 0.09, respectively). At *P*-values less than 0.1, five metabolites, including L-valine, lysine, L-tyrosine, L-isoleucine, and L-leucine, were significantly associated with RFI, accounting for 5.90 % of the phenotypic variance in RFI. Lysine, L-lactic acid, L-tyrosine, and choline were significantly associated with DMI, and these four metabolites accounted for 4.04 % of phenotypic variance in DMI. Of note, lysine and L-tyrosine were significantly associated with both RFI and DMI. Citric acid was the only metabolite that was significantly associated with ADG and accounted for 0.93 % of phenotypic variance in ADG. Four metabolites, L-glutamine, glycine, citric acid, and dimethyl sulfone, were significantly associated with MWT and accounted for 3.39 % of phenotypic variance of MWT.

**Table 1:** A summary of metabolites associated with RFI and its component traits in a multibreed population of beef cattle

Trait^1^	Metabolite^2^	*P*-value^3^	b^4^	V_m_/V_P_ (%)^5^
RFI	L-valine	6.94E-03	2.72E-03	5.90
	lysine	9.61E-03	3.96E-03	
	L-tyrosine	2.40E-02	6.65E-03	
	L-isoleucine	2.64E-02	5.80E-03	
	L-leucine	3.40E-02	3.13E-03	
DMI	lysine	1.15E-02	5.06E-03	4.04
	L-lactic acid	2.25E-02	-6.98E-05	
	L-tyrosine	2.45E-02	8.69E-03	
	choline	9.27E-02	6.69E-04	
ADG	citric acid	3.56E-02	4.31E-04	0.93
MWT	L-glutamine	1.49E-02	3.57E-02	3.39
	glycine	5.29E-02	-4.79E-03	
	citric acid	6.11E-02	-9.56E-03	
	dimethyl sulfone	9.67E-02	-2.63E-02	

^1^*RFI* residual feed intake in kg of DMI per day, *DMI* daily dry matter intake in kg per day, *ADG* average daily gain in kg, *MWT* metabolic body weight in kg

^2^The unit of metabolite concentration is µM

^3^The significance level of regression analysis is *P*-value < 0.1

^4^*b* regression coefficient

^5^*V*_*m*_*/V*_*P*_ the proportion of phenotypic variance of feed efficiency traits explained by associated metabolites (%)

### Significant SNPs and candidate genes associated with metabolites

Heritability estimates of 11 metabolites associated with feed efficiency traits were calculated (Additional file 1: Table S1). However, these estimates had large standard errors that may result from the limited number of animals utilized in this study (*n* = 493). Thus, these estimates could be used as reference information and further study may be warranted.

Metabolome-genome wide association studies were performed for the 11 metabolites associated with the feed efficiency traits. The range of *P*-value and allele substitution effect of significant SNPs, the range and average of proportion of metabolite phenotypic variance explained by each significant SNP, and the number of quantitative trait loci (QTLs) and candidate genes identified for each metabolite are summarized in Table 2. The details of the significant SNPs, including SNP position, allele substitution effect of each SNP, *P*-value and percentage of metabolite phenotypic variance explained by each SNP, for the 11 metabolites associated with feed efficiency traits are provided in Additional file 2. The candidate genes identified within a 140-kbp window of each significant SNP are shown in Additional file 3. In summary, 40, 66, 15 and 40 unique candidate genes were identified as related to RFI, DMI, ADG, and MWT, respectively (Table 3). Besides, 24 candidate genes were overlapped for RFI and DMI, 15 candidate genes were overlapped for ADG and MWT and 1 gene was common between DMI and MWT (Additional file 1: Table S2 and Additional file 4: Fig. S1).

**Table 2:** A summary of significant SNPs, the number of QTLs, and the number of candidate genes for metabolites associated with feed efficiency traits in a multibreed population of beef cattle

Metabolite^1^	*P*-value range^2^	*β* range^3^	V_SNP_/V_P_ range (%)^4^	V_SNP_/V_P_ mean (%)^5^	No. of QTLs^6^	No. of genes^7^
citric acid	1.47E-06 – 9.75E-06	-29.80 – 37.62	3.57 – 4.95	4.05	15	15
choline	4.94E-07 – 9.90E-06	-89.13 – 84.25	3.85 – 5.43	4.61	13	23
glycine	3.17E-06 – 9.54E-06	68.80 – 75.32	3.97 – 4.65	4.31	9	10
L-tyrosine	2.75E-06 – 9.40E-06	-4.20 – 7.73	3.96 – 4.49	4.11	5	2
L-isoleucine	4.21E-06 – 8.94E-06	-8.88 – 9.75	4.04 – 4.35	4.14	3	3
lysine	9.11E-09 – 9.80E-06	-17.77 – 20.56	3.88 – 7.13	4.82	15	20
L-lactic acid	2.24E-07 – 9.43E-06	-1076.62 – 1261.24	3.74 – 5.95	4.58	16	21
L-glutamine	7.37E-07 – 9.90E-06	-11.76 – 11.53	4.06 – 5.30	4.66	13	13
L-leucine	1.03E-06 – 9.30E-06	-18.12 – 17.33	3.99 – 5.04	4.40	9	12
L-valine	3.64E-06 – 9.78E-06	-25.43 – 24.38	3.73 – 4.46	4.03	8	4
dimethyl sulfone	9.44E-07 – 9.53E-06	-8.46 – 7.52	3.90 – 5.04	4.45	4	2

^1^The unit of metabolite concentration is µM

^2^The *P*-value range (minimum to maximum) of significant SNPs, the significance level is *P*-value < 1 × 10^−5^

^3^*β range* the range of allele substitution effect of each significant SNP

^4^*V*_*SNP*_*/V*_*P*_ *range* the range of metabolite phenotypic variance explained by each significant SNP (%)

^5^*V*_*SNP*_*/V*_*P*_ *mean* the average of metabolite phenotypic variance explained by each significant SNP (%)

^6^*No. of QTLs* the number of QTLs identified for each metabolite

^7^*No. of genes* the number of candidate genes identified for each metabolite

**Table 3:** Metabolites and their candidate genes associated with RFI and its component traits in a multibreed population of beef cattle

Trait^1^	Metabolite^2^	Candidate gene
RFI	L-valine	*NEDD4, PRTG, SHROOM3, XKR6*
	lysine	*BTLA, ATG3, SLC35A5, CCDC80, CD200R1L, GTPBP8, NEPRO, BOC, SPICE1, SIDT1, FGF12, HS6ST3, FRMD5, MFHAS1, STYXL2, GPA33, DAB1, OR6C75, ITPR2, SSPN*
	L-tyrosine	*ADGRF5, ADGRF1*
	L-isoleucine	*C15H11orf49, PPYR1, ANXA8L1*
	L-leucine	*SLC9A9, SYNE2, ESR2, DYNC1LI1, CD2AP, ADGRF2, ADGRF4, SHROOM3, KATNA1, LATS1, NUP43, PCMT1*
DMI	lysine	*BTLA, ATG3, SLC35A5, CCDC80, CD200R1L, GTPBP8, NEPRO, BOC, SPICE1, SIDT1, FGF12, HS6ST3, FRMD5, MFHAS1, STYXL2, GPA33, DAB1, OR6C75, ITPR2, SSPN*
	L-lactic acid	*PLSCR1, AQP9, NEDD4, PRTG, PYGO1, CUX2, NOS1, FBXO21, SPPL3, HNF1A, C17H12orf43, OASL, FOXN4, ACACB, TMEM171, FCHO2, CD247, POU2F1, MACF1, NPFFR2, SGCD*
	L-tyrosine	*ADGRF5, ADGRF1*
	choline	*HHAT, CDH8, PECAM1, MILR1, POLG2, DDX5, CEP95, ALDH3B1, NDUFS8, TCIRG1, CHKA, KMT5B, LRP5, PPP6R3, CPT1A, MRPL21, IGHMBP2, MRGPRF, CACNG2, IFT27, PVALB, BICD1, PERP*
ADG	citric acid	*SERPINE3, INTS6, ZNF667, ZNF583, USP32, CA4, ZNHIT3, MYO19, TRAF3, AMN, CDC42BPB, EDEM1, ARL8B, KLHL31, SLC28A3*
MWT	L-glutamine	*MYO16, UBE2E2, DDX56, NPC1L1, NUDCD3, CAMK2B, TRIM24, SVOPL, ATP6V0A4, PPP3CC, SORBS3, PDLIM2, CCAR2*
	glycine	*AQP9, PHLDB1, TREH, DDX6, EIF5, MARK3, SEM1, PINX1, SOX7, C8H8orf74*
	citric acid	*SERPINE3, INTS6, ZNF667, ZNF583, USP32, CA4, ZNHIT3, MYO19, TRAF3, AMN, CDC42BPB, EDEM1, ARL8B, KLHL31, SLC28A3*
	dimethyl sulfone	*ULK4, TRAK1*

^1^*RFI* residual feed intake in kg of DMI per day, *DMI* daily dry matter intake in kg per day, *ADG* average daily gain in kg, *MWT* metabolic body weight in kg

^2^The unit of metabolite concentration is µM

### Significantly enriched biological functions and gene networks for feed efficiency traits

Of the 40, 66, 15, and 40 unique candidate genes, 39, 65, 15, and 39 genes for RFI, DMI, ADG, and MWT were mapped to the IPA database for functional enrichment analyses, respectively. In summary, 24, 25, 18, and 28 significant cellular and molecular functions were identified for RFI, DMI, ADG, and MWT (*P*-value < 0.05), respectively as presented in Additional file 1: Table S3-S6. The top five enriched cellular and molecular functions with corresponding candidate genes for each feed efficiency trait are shown in Table 4. Of the top five enriched cellular and molecular functions, lipid metabolism was the biological function with the lowest *P*-value for DMI and also significantly associated with RFI and MWT (Additional file 1: Table S4 and Table S7). Molecular transport was one of the top five biological functions associated with DMI, ADG, and MWT. Small molecule biochemistry and nucleic acid metabolism were two top biological functions associated with both DMI and MWT. Among all significant biological functions, 15 biological functions were common for all four feed efficiency traits, and other biological functions shared among different feed efficiency traits are shown in Additional file 1: Table S7 and Additional file 4: Fig. S2.

**Table 4:** Five topmost significantly enriched biological functions for RFI and its component traits, and genes involved in functions

Trait^1^	Biological function	*P*-value range^2^	Genes involved in the biological function
RFI	Cellular Assembly and Organization	7.92E-05 – 4.19E-02	*ADGRF1, ADGRF5, ANXA8L1, ATG3, BOC, CD2AP, DAB1, DYNC1LI1, ESR2, FGF12, ITPR2, KATNA1, LATS1, NEDD4, SHROOM3, SPICE1, SYNE2*
	Cell Morphology	1.02E-03 – 4.02E-02	*ADGRF5, ATG3, BOC, CD2AP, ESR2, KATNA1, LATS1, NEDD4, SLC9A9, SYNE2*
	Cellular Function and Maintenance	1.02E-03 – 4.19E-02	*ADGRF1, ADGRF5, ANXA8L1, ATG3, BOC, CD2AP, DAB1, DYNC1LI1, ESR2, FGF12, ITPR2, KATNA1, NEDD4, SHROOM3, SYNE2*
	Cellular Movement	1.12E-03 – 2.87E-02	*DAB1, ESR2, KATNA1*
	Molecular Transport	1.28E-03 – 3.36E-02	*ADGRF5, DAB1, ESR2, ITPR2, LATS1, SHROOM3*
DMI	Lipid Metabolism	2.46E-04 – 2.81E-02	*ACACB, ADGRF5, ALDH3B1, AQP9, CCDC80, CHKA, CPT1A, DAB1, DDX5, HNF1A, IGHMBP2, LRP5, NOS1, PLSCR1, PVALB, SSPN*
	Molecular Transport	2.46E-04 – 2.54E-02	*ACACB, ADGRF5, AQP9, CCDC80, CD247, CHKA, CPT1A, DAB1, DDX5, FGF12, HNF1A, IFT27, IGHMBP2, ITPR2, LRP5, NEDD4, NOS1, NPFFR2, PECAM1, PLSCR1, PVALB, TCIRG1*
	Small Molecule Biochemistry	2.46E-04 – 2.81E-02	*ACACB, ADGRF5, ALDH3B1, AQP9, CCDC80, CHKA, CPT1A, DAB1, DDX5, HNF1A, HS6ST3, IGHMBP2, LRP5, NOS1, NPFFR2, PECAM1, PLSCR1, PVALB, SGCD, SSPN, TCIRG1*
	Nucleic Acid Metabolism	2.84E-04 – 2.81E-02	*ACACB, CPT1A, NOS1*
	Protein Synthesis	5.66E-04 – 1.13E-02	*ACACB, CCDC80, HNF1A, LRP5, NOS1, NPFFR2, PECAM1, SGCD*
ADG	Cell-To-Cell Signaling and Interaction	6.57E-04 – 6.57E-04	*TRAF3*
	Cellular Development	6.57E-04 – 2.34E-02	*TRAF3*
	Cellular Function and Maintenance	6.57E-04 – 2.21E-02	*AMN, ARL8B, MYO19, TRAF3*
	Cellular Growth and Proliferation	6.57E-04 – 2.34E-02	*TRAF3*
	Molecular Transport	6.57E-04 – 2.34E-02	*AMN, ARL8B, CA4, SLC28A3, TRAF3*
MWT	Molecular Transport	7.04E-04 – 4.79E-02	*AMN, AQP9, ARL8B, ATP6V0A4, CA4, CAMK2B, DDX56, DDX6, NPC1L1, PPP3CC, SLC28A3, SORBS3, TRAF3, TRAK1*
	Nucleic Acid Metabolism	7.04E-04 – 2.7E-02	*AQP9, SLC28A3*
	Small Molecule Biochemistry	7.04E-04 – 4.79E-02	*AMN, AQP9, NPC1L1, PPP3CC, SLC28A3, SORBS3, TREH*
	Cell Cycle	1.45E-03 – 2.78E-02	*ARL8B, CAMK2B, MYO19, NUDCD3, TRIM24*
	Cell Morphology	1.45E-03 – 3.36E-02	*AQP9, ARL8B, CAMK2B, NUDCD3, PDLIM2, PINX1, TRIM24*

^1^*RFI* residual feed intake in kg of DMI per day, *DMI* daily dry matter intake in kg per day, *ADG* average daily gain in kg, *MWT* metabolic body weight in kg

^2^The *P*-value range (minimum to maximum) of significant biological functions, the significance level is *P*-value < 0.05

**Fig. 1:**
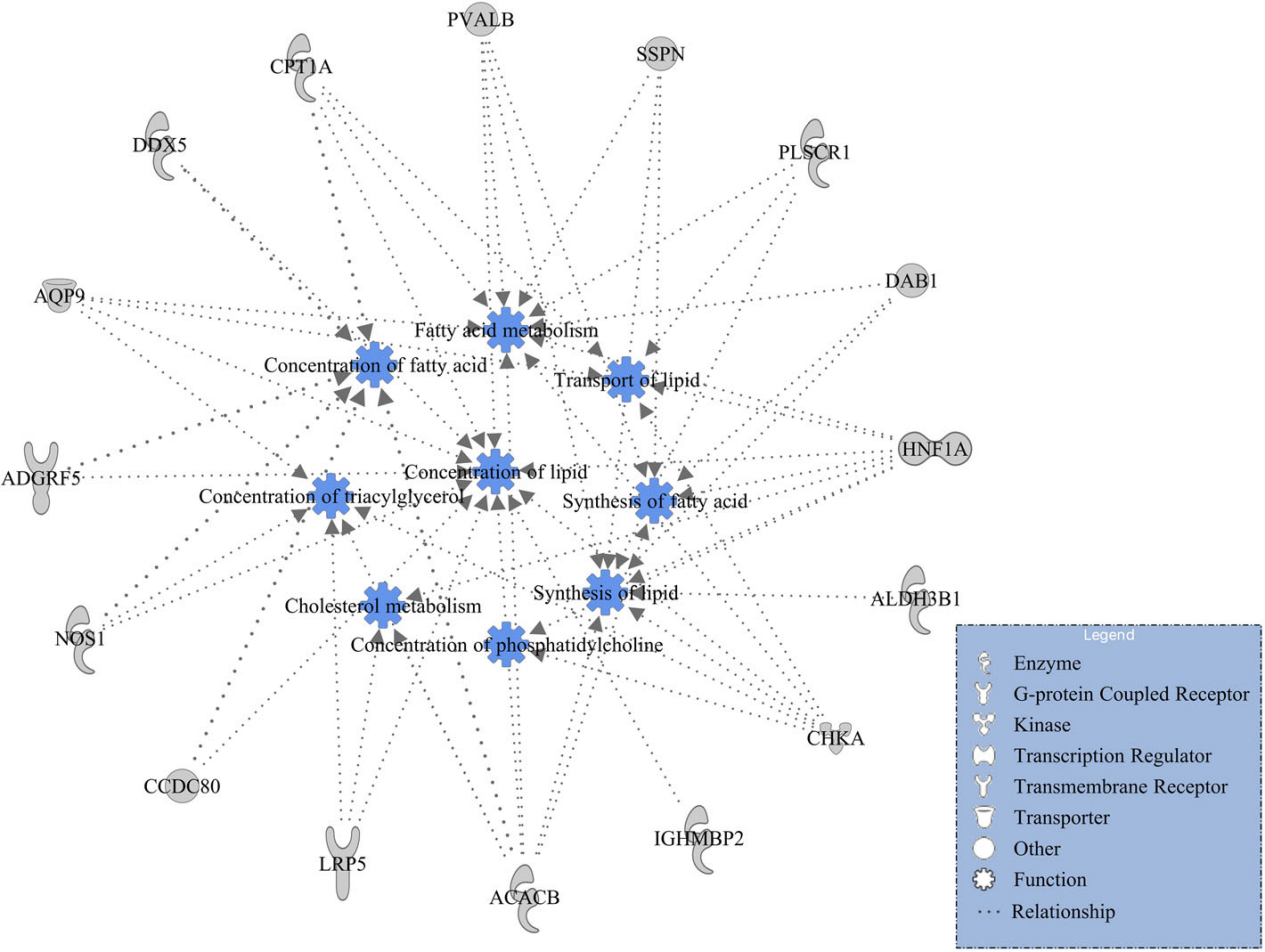
Gene network of lipid metabolism for dry matter intake (DMI)

**Fig. 2:**
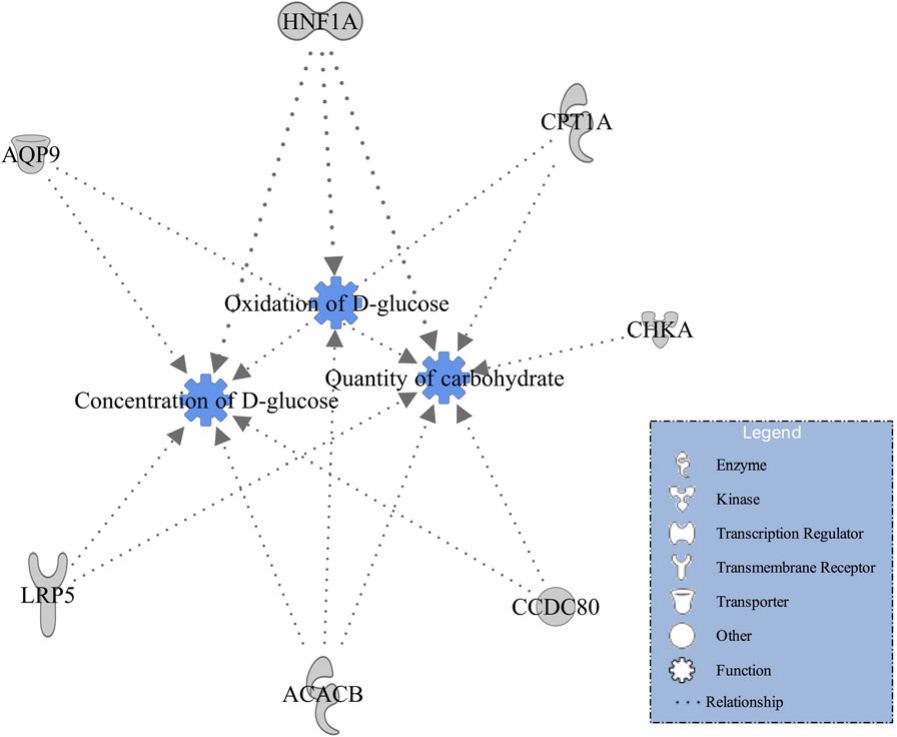
Gene network of carbohydrate metabolism for dry matter intake (DMI)

Additionally, in order to gain insight into potentially important biological functions, gene networks of lipid metabolism and carbohydrate metabolism were investigated and constructed through IPA. Within the lipid metabolism function for DMI, 16 candidate genes (*ACACB*, *ADGRF5*, *ALDH3B1*, *AQP9*, *CCDC80*, *CHKA*, *CPT1A*, *DAB1*, *DDX5*, *HNF1A*, *IGHMBP2*, *LRP5*, *NOS1*, *PLSCR1*, *PVALB,* and *SSPN*) were involved (Fig. 1). The lipid metabolism included nine subfunctions which were concentration of fatty acid, concentration of lipid, concentration of phosphatidylcholine, concentration of triacylglycerol, fatty acid metabolism, cholesterol metabolism, synthesis of fatty acid, synthesis of lipid, and transport of lipid (Fig. 1). Interestingly, seven genes (*ACACB*, *AQP9*, *CCDC80*, *CHKA*, *CPT1A*, *HNF1A,* and *LRP5*) involved in the lipid metabolism were also involved in the carbohydrate metabolism for DMI, which engaged three subfunctions including oxidation of D-glucose, concentration of D-glucose, and quantity of carbohydrate (Fig. 2).

## Discussion

### The role of metabolites in variation of feed efficiency traits

Variation in RFI and its component traits could represent differences among animals in terms of metabolic process activity. For example, a study has shown that low RFI steers tend to have more efficient metabolic process activity and are able to meet their maintenance requirement with less energy intake than high RFI steers [17]. Blood is the major highway for absorption and transportation of nutrients to the different organs and tissues, and metabolites carried by blood are directly involved in metabolic processes as substrates or products, making blood metabolites prime candidates for further studies of feed efficiency in beef cattle. Additionally, some blood metabolites have the potential to serve as biomarkers for selection of efficient beef cattle [18, 19].

In this study, 5 (L-valine, lysine, L-tyrosine, L-isoleucine, and L-leucine), 4 (lysine, L-lactic acid, L-tyrosine, and choline), 1 (citric acid) and 4 (L-glutamine, glycine, citric acid, and dimethyl sulfone) plasma metabolites were identified to be associated with RFI, DMI, ADG, and MWT, respectively (Table 1). Individual metabolites accounted for 0.59–1.50 % of the total phenotypic variance of RFI and its component traits. The results suggest that the feed efficiency traits could be associated with many metabolites with small effects. However, the identified metabolites associated with the feed efficiency traits in this study may require validation in independent beef cattle populations especially as a more relaxed threshold (*P*-value < 0.1) was used. Furthermore, we would like to highlight that only 31 metabolites were detected by the targeted method of NMR used in the current study. We therefore recommend that metabolomic profiles with more metabolites should be investigated in future with larger samples in order to identify more metabolites that are associated with RFI or its component traits.

To date, several metabolomic studies have attempted to identify relationships between serum or plasma metabolite levels and RFI in beef cattle [18, 20, 21]. We found good agreement between the results from those studies and the current study. In the current study, valine, lysine, tyrosine, and leucine showed higher concentrations in beef cattle with high RFI than those with low RFI. In line with our results, Karisa et al. [18] and Foroutan et al. [20] observed higher concentrations of valine, lysine, and tyrosine in beef cattle with high RFI as compared to those with low RFI. Similarly, Jorge-Smeding et al. reported that concentrations of valine and lysine were decreased in heifers with low RFI [21]. Additionally, Foroutan et al. reported the concentration of leucine was higher in high-RFI beef cattle [20], which is consistent with our results. The consistency of results from different studies suggests that these metabolites have the potential to be used as biomarkers for feed efficiency.

It is worth noting that, the three metabolites (isoleucine, leucine, and valine) associated with RFI are three essential branched-chain amino acids. These three metabolites share the first enzymatic steps in their oxidative pathways, including a reversible transamination followed by an irreversible oxidative decarboxylation to coenzyme-A derivatives [22]. The respective oxidative pathways subsequently diverge and at the final steps yield acetyl- and/or propionyl-CoA that enter the tricarboxylic acid cycle (TCA cycle) [22]. For animals, the TCA cycle is the main energy producing (mainly from carbohydrates and fatty acids) metabolic pathway [23], and some of the processes of the TCA cycle pathway have been previously reported to be associated with feed efficiency in beef cattle [18] and pigs [24]. Additionally, in this study, citric acid was the only metabolite that was significantly associated with ADG and was overlapping for ADG and MWT. Citric acid is an important intermediate in the TCA cycle [23] indicating a potential relationship between the TCA cycle related metabolic processes and feed efficiency traits. Interestingly, two other metabolites (lysine and L-tyrosine) were identified as associated with both RFI and DMI in this study. In the current study, we observed that the concentrations of lysine and L-tyrosine were significantly positively correlated (*r* = 0.29, *P*-value < 0.001). A previous study reported a higher positive correlation (*r* > 0.75, *P*-value < 0.001) between lysine and tyrosine [24]. The association of lysine and L-tyrosine with both RFI and DMI could be due to the significant positive correlation between lysine and tyrosine and the fact that RFI has a high and positive genetic correlation with DMI (*r*_*g*_ = 0.66±0.11 to 0.75±0.10) [11, 25]. Furthermore, lysine and tyrosine were reported as important amino acids involved in some important metabolic processes in beef cattle, such as amino acid metabolism and urea cycle [21], further supporting them as potential biomarkers for feed efficiency traits.

### Candidate genes, enriched molecular functions and gene networks for feed efficiency traits

In this study, we identified 40, 66, 15, and 40 unique candidate genes as related to RFI, DMI, ADG, and MWT respectively via integrative analyses of regression analyses and mGWAS (Table 3). In a previous study, Zhang et al. performed GWAS based on imputed whole genome sequence variants for RFI, DMI, ADG, and MWT using 7,500 beef cattle and reported 596, 268, 179, and 532 candidate genes for RFI, DMI, ADG, and MWT, respectively [12]. Comparing their results with those in this study, we found 10, 23, 6, and 7 candidate genes in common between the two studies for RFI, DMI, ADG, and MWT, respectively (Additional file 1: Table S8). These overlapping genes indicated that metabolites are potentially important intermediate phenotypes between candidate genes and feed efficiency traits. Additionally, results from our study provide more knowledge and better understanding of how the previously identified candidate genes exert their influence on the variability of RFI and its component traits via intermediate phenotype metabolites. For instance, Zhang et al. reported that some genes were associated with more than one trait such as, *ADGRF1* and *ADGRF5* which were associated with both RFI and DMI [12]. However, the potential mechanism of how these genes could influence the two traits remained unclear. According to the results of the current study, these two genes were both associated with L-tyrosine as a common metabolite which was associated with RFI and DMI (Table 3). Similarly, according to Zhang et al., *SLC28A3* was associated with ADG and MWT [12], and our results showed this gene was associated with citric acid as a common metabolite which was associated with ADG and MWT (Table 3). Interestingly, Zhang et al. identified *ADGRF1*, *ADGRF5*, *GTPBP8,* and *NEPRO* as associated with both RFI and DMI [12] and the same genes for RFI and DMI were identified in the current study. However, the results of this study indicated that the molecular background of these associations might be different. L-tyrosine might explain the associations of *ADGRF1*, *ADGRF5* with RFI and DMI, because we identified that *ADGRF1* and *ADGRF5* were associated with L-tyrosine which was a metabolite associated with both RFI and DMI. As for *GTPBP8* and *NEPRO*, both genes were associated with another common metabolite called lysine that was identified to be associated with both RFI and DMI in the current study. Additionally, we observed that certain genes might be associated with the same feed efficiency trait through different metabolites. For example, *SHROOM3* was associated with L-valine and L-leucine and these two metabolites were associated with RFI (Table 3). Our study also showed that certain genes could be associated with different feed efficiency traits through different metabolites. For example, *AQP9* was associated with DMI and MWT through L-lactic acid and glycine, respectively (Table 3). Therefore, our integrative analyses of feed efficiency traits, metabolites, and whole genome sequence variants will enhance our understanding on genetic influence of feed efficiency traits in beef cattle.

Some candidate genes identified for feed efficiency traits in the current study have been reported in our previous transcriptomic studies involving animals related to those used in the current study [26, 27]. For instance, *CCDC80* was reported as a differentially expressed gene between beef steers with divergent RFI [26]. Additionally, *CCDC80*, *CUX2,* and *ALDH3B1* were differentially expressed in the liver of beef steers for DMI, and *SERPINE3* was a differentially expressed gene for ADG [27]. Our current study identified the same genes associated with these traits through integrating metabolites (Table 3). Indeed, *CCDC80*, *CUX2*, *ALDH3B1,* and *SERPINE3* were associated with lysine, L-lactic acid, choline, and citric acid, respectively. Therefore, our results potentially provide further insight into how these differentially expressed genes affect the feed efficiency traits in beef cattle. It is worth noting that *CUX2* has also been reported to be associated with DMI in the American [13] and Canadian beef population [12]. Therefore, these genes identified as associated with the same feed efficiency traits using genomic, transcriptomic and metabolomic data suggest the importance of these genes in influencing feed efficiency traits in beef cattle. Furthermore, some differentially expressed genes may affect RFI by influencing metabolites associated with its component traits (DMI, ADG, and MWT). For example, *TCIRG1*, *AMN*, and *AQP9* were reported as differentially expressed genes in high- and low-RFI beef cattle [28, 29] and these three genes were identified to be respectively associated with DMI, ADG, and MWT through different metabolites in this study.

Identification of enriched molecular processes, pathways and gene networks associated with feed efficiency traits using candidate genes from these different omics studies shed some light on underlying biological mechanism and gene interactions for complex traits. For the five topmost biological functions associated with RFI in the current study, cellular assembly and organization, cell morphology, cellular function and maintenance, and molecular transport were four biological functions that overlapped with the five topmost biological functions reported by Zhang et al. for RFI [12]. Lipid metabolism, small molecule biochemistry, and nucleic acid metabolism were three common top biological functions for DMI in the two studies. Lipid metabolism and small molecule biochemistry were also identified as two of the five topmost biological functions in our previous transcriptomic study for DMI in beef cattle [27]. Molecular transport, small molecule biochemistry, and cell morphology were three overlapping top biological functions for MWT in Zhang et al. [12] and in this study. These three biological functions were also top biological functions for MWT in our previous transcriptomic study [27]. For ADG, cell-to-cell signaling and interaction was a common top biological functions in Zhang et al. [12], Mukiibi et al. [27] and in the current study. Our results and those reported by previous studies indicated the overlapping top five biological functions have a potentially important relationship with feed efficiency traits in beef cattle. These important functions could further help to prioritize candidate genes and related functional SNPs associated with phenotypes.

Additionally, we would like to note that attention should be paid to nutrient or energy metabolic processes, such as lipid metabolism, since several studies have reported its potential role in feed efficiency related to DMI and RFI [11, 12, 26, 27, 29–34]. Nkrumah et al. [34] and Mao et al. [11] reported that more efficient beef cattle tended to have less backfat and slightly less marbling. Transcriptomic studies reported that more efficient beef cattle were associated with differentially expressed genes related to reducing lipid metabolism in liver [26, 27], implying an important relationship between lipid metabolism and feed efficiency. Weber et al. identified differentially expressed genes in multiple tissues (pituitary, skeletal muscle, liver, visceral adipose, and duodenum) of beef cattle with divergent RFI, and their pathway analyses showed that many of the differentially expressed genes were involved in the immune system and fat metabolism [29]. In this study, lipid metabolism was the most significant biological functions for DMI and also significantly associated with RFI and MWT. Lipid metabolism was identified as one of the top biological functions for ADG in previous studies [12, 27] but it was not shown in the current study, which is likely due to limitations of relatively small number of metabolites analyzed. In addition, of the candidate genes identified for the metabolites, there is limited knowledge on how candidate genes influence the respective plasma metabolite levels. For instance, enzyme choline kinase alpha is encoded by *CHKA* [35]. In the biosynthesis pathway of phosphatidylcholine, the enzyme can catalyze the phosphorylation of choline to phosphocholine [36, 37]. However, little is known on how concentrations of choline vary among animals due to their gene variants. Nevertheless, our integrative study of feed efficiency, blood metabolites, and DNA variants has provided additional insight into relationships between gene functionalities, metabolites, and feed efficiency traits, which may help develop strategies to enhance genomic prediction of feed efficiency traits with incorporation of metabolite data.

## Conclusions

This study combined genomic, metabolomic and phenotypic data to investigate molecules and biological functions/processes related to feed efficiency in beef cattle. Several plasma metabolites associated with RFI and its component traits were identified, and some of metabolites showed the potential to serve as biomarkers for feed efficiency in beef cattle. Multiple candidate genes were identified as associated with RFI and its component traits based on the results of regression analyses between feed efficiency traits and metabolites, and mGWAS. Gene functional enrichment analyses indicated that lipid metabolism may have an important role in feed efficiency. Our findings showed good consistency with previous metabolomic studies and GWAS studies for feed efficiency and also added more information regarding biological mechanisms of feed efficiency. Therefore, this integrative method could enhance the understanding of genetic influence, metabolites and biological functions/processes involved in feed efficiency traits, which could lead to improvement of genomic prediction accuracy via incorporating metabolite data.

## Methods

### Animal population, data collection of feed efficiency traits and metabolites

All animals in this study were cared for according to the guidelines of the Canadian Council on Animal Care (1993). The population of animals was obtained from the Phenomic Gap Project that aimed to generate phenotypes of feed efficiency, carcass and meat quality as well as genomic data for Canadian crossbred beef animals [38]. Details of animal management, the herd, and animal breeds were previously described [12, 39–41]. In summary, the population used in this study consisted of 493 crossbred bulls (*n* = 93), heifers (*n* = 125) and steers (*n* = 275) that were born between 2002 and 2011. These animals were from five different commercial herds and they were tested in feedlots from 2003 to 2012 [38]. The major breed components were primarily Charolais (*n* = 73), Hereford-Angus crosses (*n* = 191) and a Beefbooster composite breed (predominantly Charolais-based, *n* = 229). The GrowSafe system (GrowSafe Systems Ltd., Airdrie, Alberta, Canada) was used to measure the feed intake of finishing calves at the feeding test station for a period of 76 to 112 days. Serial body weights (BW) in kg were measured for each animal at the beginning and end of the test and at approximately 14-day intervals during the test. The daily DMI in kg was calculated as an average of dry matter intake over the test period and further standardized according to the energy content of the diet. The initial BW in kg at the start of the feeding test and the ADG in kg were derived from a linear regression of the serial BW measurements against time (day) [11, 12, 34, 42]. The MWT in kg was calculated as midpoint BW^0.75^ while the midpoint BW was computed as the sum of the initial BW in kg and the product of ADG multiplied by half of the days on test [11, 12, 34, 42]. The RFI in kg of DMI per day was computed as the difference between the standardized daily DMI and the expected DMI that was predicted based on animal ADG and MWT [7]. Blood samples were collected from all animals by jugular venipuncture in the early morning on the first day of feedlot tests and immediately frozen at -80 °C for storage. These blood samples were used to quantify metabolites using nuclear magnetic resonance (NMR) spectroscopy. The procedure of metabolite quantification using NMR was previously described by Li et al. [39]. Thirty-one metabolites and their concentration levels (µM) were quantified from plasma. Blood samples were also used to extract DNA for genotyping using the Illumina BovineSNP50 v2 BeadChip (Illumina Inc., CA, USA).

### SNP genotype imputation, quality control and population admixture analyses

Theoretically, a higher marker density could improve the power of GWAS to identify significant SNPs, therefore, the 50 K genotypes were imputed to whole genome sequence variants using Beagle 5.1 software [43]. The SNP imputation for animals used in this study was completed using a step-wise approach as described by Zhang et al. [12] and Wang et al. [44] based on the latest genome assembly ARS-UCD 1.2. After the imputation, 53,258,178 SNPs and indels (they are all termed SNPs for simplicity) on 29 autosomes were obtained. Quality control for imputed whole genome sequence variants was performed to exclude DNA variants based on the following criteria: SNPs on 29 autosomes that had an imputation accuracy < 0.95, minor allele frequency < 0.05, and failed to pass the Hardy-Weinberg equilibrium test (*P*-value < 0.0001). Finally, a total of 10,488,742 SNPs remained after quality control and were used in further analyses.

Breed composition of each animal was predicted based on the 50 K genotypes using ADMIXTURE software to account for population stratification [45, 46]. In order to find the best possible number of ancestors or breeds (K value), a 5-fold cross-validation procedure was performed as described in Zhang et al. [12]. The breed composition prediction had the smallest cross-validation error when the value of K = 6. The most accurate breed composition was then obtained for each individual and presented in Additional file 1: Table S9.

### Data consolidation, quality control for feed efficiency traits and metabolites

The variation in feed efficiency traits and metabolites could be affected by multiple systematic effects. A linear regression model implemented in R statistical software was used to assess the significant systematic effects that were associated with feed efficiency traits or metabolites. Animal type (bull, heifer, steer), birth year, herd, feedlot pen, age at the feedlot test, and breeding composition were found to be the significantly associated factors for both the feed efficiency traits and metabolites (*P*-value < 0.05). Therefore, phenotypic values of the feed efficiency traits and metabolites were pre-adjusted for the above factors using liner regression models. Residuals with more or less than 3 standard deviations from the mean of residuals were considered as outliers and were excluded. Additional file 1: Table S10 provides descriptive statistics of phenotypic data on the feed efficiency traits and metabolites.

### Regression analyses between feed efficiency traits and metabolites and metabolome-genome wide association studies

After quality control and pre-adjustment of phenotypic data, regression analyses were conducted to identify associations between four feed efficiency traits and thirty-one metabolites using R statistical software. A feed efficiency trait and a metabolite were considered to be significantly associated when a *P*-value < 0.1 of the regression analyses was observed. This step was intended to determine the relationship between feed efficiency traits and metabolites. The mGWAS (metabolome-genome wide association studies) were performed for metabolites that were significantly associated with the feed efficiency traits using the mlma (mixed linear model association) option as implemented in the GCTA package [47] based on the following linear mixed model:

$${y}_{ij}=\mu +{b}_{j}{x}_{ij}+{a}_{ij}+{e}_{ij}$$

where ${y}_{ij}$ is the adjusted metabolite value of the $i$th animal with the $j$th SNP (i.e. the $ij$th animal), ${b}_{j}$ is the allele substitution effect of the $j$th SNP, ${x}_{ij}$ is the $j$th SNP genotype of animal $i$ coded as 0, 1, 2 for genotypes ${A}_{1}{A}_{1}$, ${A}_{1}{A}_{2}$, and ${A}_{2}{A}_{2}$, respectively, ${a}_{ij}$ is the additive polygenic effect of the $ij$th animal $\tilde N\left(0,{G}{\sigma }_{a}^{2}\right)$, and ${e}_{ij}$ is the random residual effect $\tilde N\left(0, {I}{\sigma }_{e}^{2}\right)$. The genomic relationship matrix ${G}$ was derived based on total filtered SNP markers (i.e. 10,488,742 SNPs) as described by Yang et al. [48], which is essentially the same as the second VanRaden formulation [49]. The same ${G}$ matrix was used to estimate variance components and heritability of metabolites via restricted maximum likelihood (REML) as implemented in the GCTA package.

The SNPs with *P*-value < 1 × 10^−5^ were classified to be significantly associated with the metabolite according to the recommendation of The Wellcome Trust Case Control Consortium [50]. The phenotypic variance of the metabolite explained by each significant SNP was calculated by $\frac{2pq{\beta }^{2}}{{S}^{2}}\text{*}100\text{\%}$, where $p$ and $q$ denote the SNP allele frequency of ${ A}_{1 }$ and ${A}_{2}$, respectively; $\beta$ is the SNP allele substitution effect that was estimated by generalized least square and the significance of SNP allele substitution effect was conducted via a generalized least square F-test as implemented in the GCTA package; $2pq{\beta }^{2}$ is the additive variance of the SNP, and ${S}^{2}$ is the phenotypic variance of the metabolite.

### Identification of candidate genes and functional enrichment analyses for feed efficiency traits

To identify candidate genes for concentration of each metabolite, a 140-kbp window (70-kbp upstream and 70-kbp downstream) of each significant SNP was surveyed based on SNP annotation information from ARS-UCD 1.2 bovine genome assembly from the Ensembl BioMart database (accessed on 02 February, 2021). The 70-kbp was the chromosomal length within which a high linkage disequilibrium phase correlation (${r}^{2}$> 0.77) was maintained across a sample of Canadian beef cattle breeds [51]. Small nucleolar RNA and microRNA were excluded because we are interested in protein coding genes. Then candidate genes (Entrez gene IDs) of all metabolites that were associated with the feed efficiency traits (RFI, DMI, ADG, or MWT) as identified in the regression analyses were combined and imported into the Ingenuity Pathway Analysis software (accessed on 02 February, 2021) (IPA; www.Ingenuity.com) to predict the enriched biological functions and gene networks for feed efficiency traits. Biological functions were considered significantly enriched if the *P*-value for the overlap comparison test between the input gene list and the knowledge base of IPA for a given biological function was less than 0.05. In order to provide insight into cellular and molecular functions associated with feed efficiency traits, gene networks for some significant biological functions were constructed in IPA.

## Supplementary Information

**Additional file 1: Table S1.** Additive genetic variances and heritability estimates for 11 metabolites based on the imputed whole genome sequence (WGS) variants in a beef cattle multibreed population; **Table S2.** Uniquely common candidate genes for feed efficiency traits in a beef cattle multibreed population; **Table S3.** Enriched biological functions significantly associated with RFI in a beef cattle multibreed population; **Table S4.** Enriched biological functions significantly associated with DMI in a beef cattle multibreed population; **Table S5.** Enriched biological functions significantly associated with ADG in a beef cattle multibreed population; **Table S6.** Enriched biological functions significantly associated with MWT in a beef cattle multibreed population; **Table S7.** Uniquely common biological functions for feed efficiency traits in a beef cattle multibreed population; **Table S8.** The comparison of candidate genes between the current study and Zhang et al; **Table S9.** Genomic breed composition for animals; **Table S10.** Descriptive statistics of phenotypic data on feed efficiency traits and metabolites.

**Additional file 2.** This file contains a summary of SNPs significantly associated with the 11 metabolites.

**Additional file 3. **This file contains a summary of candidate genes for the 11 metabolites.

**Additional file 4: Figure S1.** Uniquely common candidate genes for feed efficiency traits in a beef cattle multibreed population; **Figure S2.** Uniquely common biological functions for feed efficiency traits RFI, DMI, ADG, MWT in a beef cattle multibreed population

## Data Availability

The dataset supporting the results of this article are included within the article and its additional files. Whole genome sequence datasets generated and/or analyzed during the current study for imputation are available from the NCBI SRA database under BioProjects PRJNA176557 and PRJNA256210. The original genotype and phenotype data sets are available for non-commercial purposes from GP or CL following the execution of a materials transfer agreement.

## Abbreviations

ADG: Average daily gain
BW: Body weight
DMI: Daily dry matter intake
DNA: Deoxyribonucleic acid
GWAS: Genome-wide association studies
IPA: Ingenuity Pathway Analysis software
mGWAS: Metabolome-genome wide association studies
mlma: Mixed linear model association
MWT: Metabolic body weight
NMR: Nuclear magnetic resonance
QTL: Quantitative trait locus
REML: Restricted maximum likelihood
RFI: Residual feed intake
SNP: Single nucleotide polymorphism
TCA cycle: Tricarboxylic acid cycle

## References

[1] Ahola JK, Hill RA. Input Factors Affecting Profitability: A Changing Paradigm and a Challenging Time. In: Feed Efficiency in the Beef Industry. Hoboken, New Jersey: Wiley-Blackwell; 2012. p. 7–19.

[2] Ramsey R, Doye D, Ward C, McGrann J, Falconer L, Bevers S (2005). Factors affecting beef cow-herd costs, production, and profits. J Agric Appl Econ.

[3] Nielsen MK, MacNeil MD, Dekkers JCM, Crews DH, Rathje TA, Enns RM (2013). Life-cycle, total-industry genetic improvement of feed efficiency in beef cattle: Blueprint for the Beef Improvement Federation11The development of this commentary was supported by the Beef Improvement Federation. Professional Animal Scientist.

[4] Archer JA, Barwick SA, Graser HU (2004). Economic evaluation of beef cattle breeding schemes incorporating performance testing of young bulls for feed intake. Aust J Exp Agric.

[5] Gerber PJ, Steinfeld H, Henderson B, Mottet A, Opio C, Dijkman J, et al. Tackling climate change through livestock – A global assessment of emissions and mitigation opportunities. Rome, Italy: Food and Agriculture Organization of the United Nations (FAO); 2013.

[6] Hegarty RS, Goopy JP, Herd RM, McCorkell B (2007). Cattle selected for lower residual feed intake have reduced daily methane production. J Anim Sci.

[7] Koch RM, Swiger LA, Chambers D, Gregory KE (1963). Efficiency of feed use in beef cattle. J Anim Sci.

[8] Herd RM, Bishop SC (2000). Genetic variation in residual feed intake and its association with other production traits in British Hereford cattle. Livest Prod Sci.

[9] Arthur PF, Renand G, Krauss D (2001). Genetic and phenotypic relationships among different measures of growth and feed efficiency in young Charolais bulls. Livest Prod Sci.

[10] Nkrumah JD, Okine EK, Mathison GW, Schmid K, Li C, Basarab JA (2006). Relationships of feedlot feed efficiency, performance, and feeding behavior with metabolic rate, methane production, and energy partitioning in beef cattle. J Anim Sci.

[11] Mao F, Chen L, Vinsky M, Okine E, Wang Z, Basarab J (2013). Phenotypic and genetic relationships of feed efficiency with growth performance, ultrasound, and carcass merit traits in Angus and Charolais steers. J Anim Sci.

[12] Zhang F, Wang Y, Mukiibi R, Chen L, Vinsky M, Plastow G, et al. Genetic architecture of quantitative traits in beef cattle revealed by genome wide association studies of imputed whole genome sequence variants: I: feed efficiency and component traits. BMC Genomics. 2020;21:36. 10.1186/s12864-019-6362-1 PMC6956504 31931702

[13] Seabury CM, Oldeschulte DL, Saatchi M, Beever JE, Decker JE, Halley YA (2017). Genome-wide association study for feed efficiency and growth traits in U.S. beef cattle. BMC Genomics.

[14] Bolormaa S, Hayes BJ, Savin K, Hawken R, Barendse W, Arthur PF (2011). Genome-wide association studies for feedlot and growth traits in cattle. J Anim Sci.

[15] Abo-Ismail MK, Vander Voort G, Squires JJ, Swanson KC, Mandell IB, Liao X (2014). Single nucleotide polymorphisms for feed efficiency and performance in crossbred beef cattle. BMC Genet.

[16] Fontanesi L (2016). Metabolomics and livestock genomics: Insights into a phenotyping frontier and its applications in animal breeding. Anim Front.

[17] Richardson EC, Herd RM (2004). Biological basis for variation in residual feed intake in beef cattle. 2. Synthesis of results following divergent selection. Aust J Exp Agric.

[18] Karisa BK, Thomson J, Wang Z, Li C, Montanholi YR, Miller SP (2014). Plasma metabolites associated with residual feed intake and other productivity performance traits in beef cattle. Livest Sci.

[19] Novais FJ, Pires PRL, Alexandre PA, Dromms RA, Iglesias AH, Ferraz JBS (2019). Identification of a metabolomic signature associated with feed efficiency in beef cattle. BMC Genomics.

[20] Foroutan A, Fitzsimmons C, Mandal R, Berjanskii MV, Wishart DS (2020). Serum metabolite biomarkers for predicting residual feed intake (RFI) of young angus bulls. Metabolites.

[21] Jorge-Smeding E, Renand G, Centeno D, Pétéra M, Durand S, Polakof S, et al. Metabolomics reveals changes in urea cycle associated to residual feed intake in growing heifers. In: EAAP Scientific Series. Wageningen, Netherlands: Wageningen Academic Publishers; 2019. p. 231–2.

[22] Manoli I, Venditti CP (2016). Disorders of branched chain amino acid metabolism. Transl Sci Rare Dis.

[23] Akram M (2014). Citric acid cycle and role of its intermediates in metabolism. Cell Biochem Biophys.

[24] Wang X, Kadarmideen HN. Metabolomics analyses in high-low feed efficient dairy cows reveal novel biochemical mechanisms and predictive biomarkers. Metabolites. 2019;9:151. 10.3390/metabo9070151 PMC6680417 31340509

[25] Ceacero TM, Mercadante MEZ, Cyrillo JNDSG, Canesin RC, Bonilha SFM, De Albuquerque LG. Phenotypic and genetic correlations of feed efficiency traits with growth and carcass traits in nellore cattle selected for postweaning weight. PLoS One. 2016;11:e0161366. 10.1371/journal.pone.0161366 PMC4990259 27537268

[26] Mukiibi R, Vinsky M, Keogh KA, Fitzsimmons C, Stothard P, Waters SM (2018). Transcriptome analyses reveal reduced hepatic lipid synthesis and accumulation in more feed efficient beef cattle. Sci Rep.

[27] Mukiibi R, Vinsky M, Keogh K, Fitzsimmons C, Stothard P, Waters SM (2019). Liver transcriptome profiling of beef steers with divergent growth rate, feed intake, or metabolic body weight phenotypes. J Anim Sci.

[28] Tizioto PC, Coutinho LL, Decker JE, Schnabel RD, Rosa KO, Oliveira PSN (2015). Global liver gene expression differences in Nelore steers with divergent residual feed intake phenotypes. BMC Genomics.

[29] Weber KL, Welly BT, Van Eenennaam AL, Young AE, Porto-Neto LR, Reverter A (2016). Identification of gene networks for residual feed intake in Angus cattle using genomic prediction and RNA-seq. PLoS One..

[30] Alexandre PA, Kogelman LJA, Santana MHA, Passarelli D, Pulz LH, Fantinato-Neto P (2015). Liver transcriptomic networks reveal main biological processes associated with feed efficiency in beef cattle. BMC Genomics.

[31] Chen Y, Gondro C, Quinn K, Herd RM, Parnell PF, Vanselow B (2011). Global gene expression profiling reveals genes expressed differentially in cattle with high and low residual feed intake. Anim Genet.

[32] McKenna C, Porter RK, Keogh KA, Waters SM, McGee M, Kenny DA (2018). Residual feed intake phenotype and gender affect the expression of key genes of the lipogenesis pathway in subcutaneous adipose tissue of beef cattle. J Anim Sci Biotechnol.

[33] Higgins MG, Kenny DA, Fitzsimons C, Blackshields G, Coyle S, McKenna C (2019). The effect of breed and diet type on the global transcriptome of hepatic tissue in beef cattle divergent for feed efficiency. BMC Genomics.

[34] Nkrumah JD, Basarab JA, Wang Z, Li C, Price MA, Okine EK (2007). Genetic and phenotypic relationships of feed intake and measures of efficiency with growth and carcass merit of beef cattle. J Anim Sci.

[35] Hosaka K, Tanaka S, Nikawa J, ichi, Yamashita S (1992). Cloning of a human choline kinase cDNA by complementation of the yeast *cki* mutation. FEBS Lett.

[36] Aoyama C, Liao H, Ishidate K (2004). Structure and function of choline kinase isoforms in mammalian cells. Prog Lipid Res.

[37] Lacal JC (2001). Choline kinase: a novel target for antitumor drugs. IDrugs.

[38] McKeown L, Aalhus J, Larsen I, Stothard P, Wang Z. Bridging the “Phenomic Gap”: Creation of a database containing phenotypes and genotypes for economically important traits for beef cattle. Edmonton, Canada. Final Report to the Alberta Livestock and Meat Agency; 2013.

[39] Li J, Akanno EC, Valente TS, Abo-Ismail M, Karisa BK, Wang Z (2020). Genomic heritability and genome-wide association studies of plasma metabolites in crossbred beef cattle. Front Genet.

[40] Akanno EC, Plastow G, Woodward BW, Bauck S, Okut H, Wu XL (2014). Reliability of molecular breeding values for Warner-Bratzler shear force and carcass traits of beef cattle - An independent validation study. J Anim Sci.

[41] Abo-Ismail MK, Lansink N, Akanno E, Karisa BK, Crowley JJ, Moore SS (2018). Development and validation of a small SNP panel for feed efficiency in beef cattle. J Anim Sci.

[42] Lu D, Miller S, Sargolzaei M, Kelly M, Vander Voort G, Caldwell T (2013). Genome-wide association analyses for growth and feed efficiency traits in beef cattle. J Anim Sci.

[43] Browning BL, Zhou Y, Browning SR (2018). A one-penny imputed genome from next-generation reference panels. Am J Hum Genet.

[44] Wang Y, Zhang F, Mukiibi R, Chen L, Vinsky M, Plastow G, et al. Genetic architecture of quantitative traits in beef cattle revealed by genome wide association studies of imputed whole genome sequence variants: II: carcass merit traits. BMC Genomics. 2020;21:38. 10.1186/s12864-019-6273-1 PMC6958779 31931697

[45] Alexander DH, Novembre J, Lange K (2009). Fast model-based estimation of ancestry in unrelated individuals. Genome Res.

[46] Hellwege JN, Keaton JM, Giri A, Gao X, Velez Edwards DR, Edwards TL (2017). Population stratification in genetic association studies. Curr Protoc Hum Genet..

[47] Yang J, Lee SH, Goddard ME, Visscher PM (2011). GCTA: A tool for genome-wide complex trait analysis. Am J Hum Genet.

[48] Yang J, Zaitlen NA, Goddard ME, Visscher PM, Price AL (2014). Advantages and pitfalls in the application of mixed-model association methods. Nat Genet.

[49] VanRaden PM (2008). Efficient methods to compute genomic predictions. J Dairy Sci.

[50] Burton PR, Clayton DG, Cardon LR, Craddock N, Deloukas P, Duncanson A (2007). Genome-wide association study of 14,000 cases of seven common diseases and 3,000 shared controls. Nature.

[51] Lu D, Sargolzaei M, Kelly M, Li C, Vander Voort G, Wang Z, et al. Linkage disequilibrium in Angus, Charolais, and Crossbred beef cattle. Front Genet. 2012;3:152. 10.3389/fgene.2012.00152 PMC3418579 22912646

[52] Kilkenny C, Browne WJ, Cuthill IC, Emerson M, Altman DG (2010). Improving bioscience research reporting: the ARRIVE guidelines for reporting animal research. PLoS Biol.

