# Supplementary material for: Identification of candidate genes and enriched biological functions for feed efficiency traits by integrating plasma metabolites and imputed whole genome sequence variants in beef cattle
Source: BMC Genomics. 2021 Nov 15;22:823. doi: 10.1186/s12864-021-08064-5 (PMC8591823; doi:10.1186/s12864-021-08064-5)
Supplement: Supplementary file 4 — Additional file 4: Figure S1. Uniquely common candidate genes for feed efficiency traits in a beef cattle multibreed population; Figure S2. Uniquely common biological functions for feed efficiency traits RFI, DMI, ADG, MWT in a beef cattle multibreed population [file 12864_2021_8064_MOESM4_ESM.pdf]

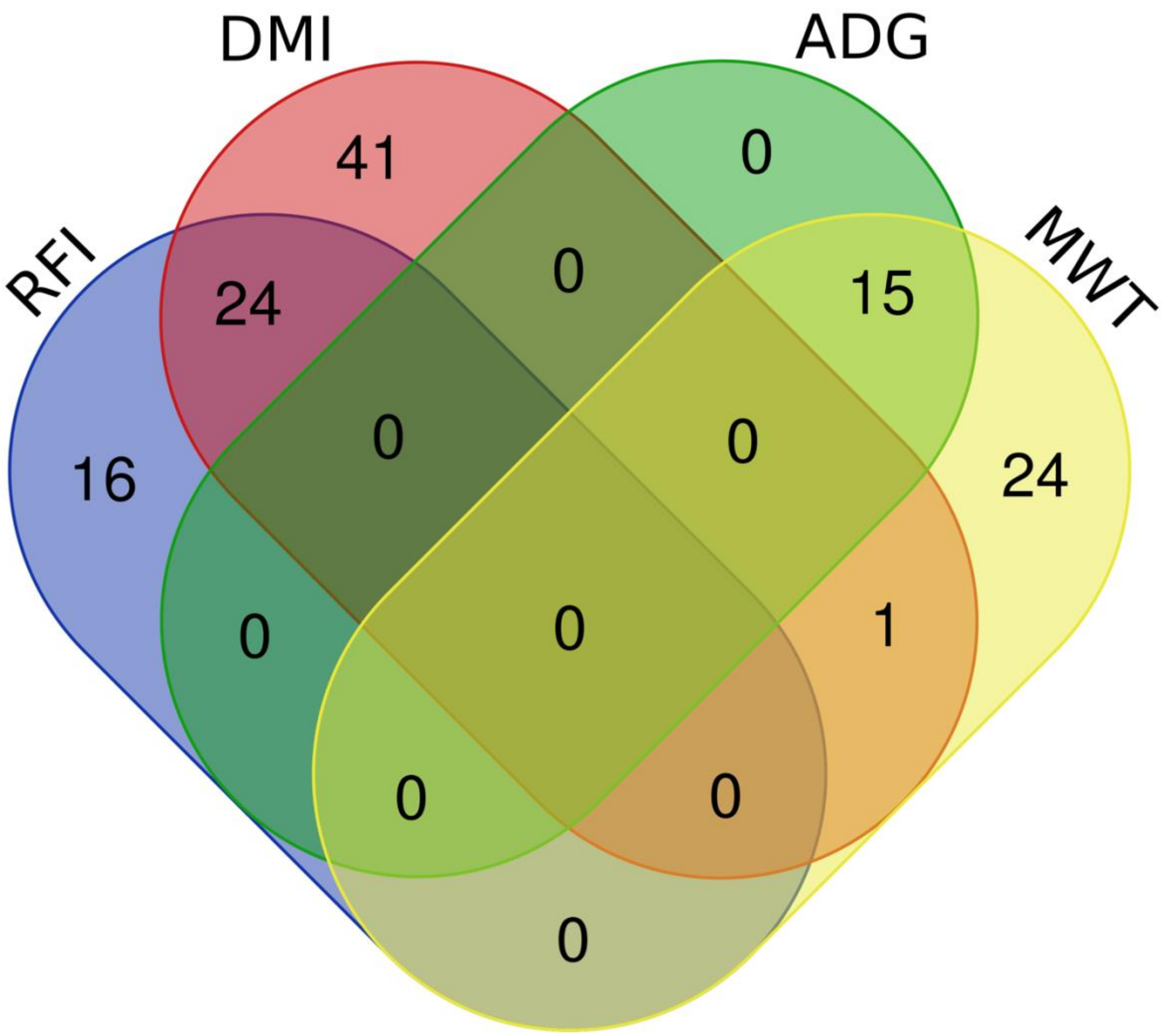

Figure S1 Uniquely common candidate genes for feed efficiency traits RFI, DMI, ADG, MWT in a beef cattle multibreed population

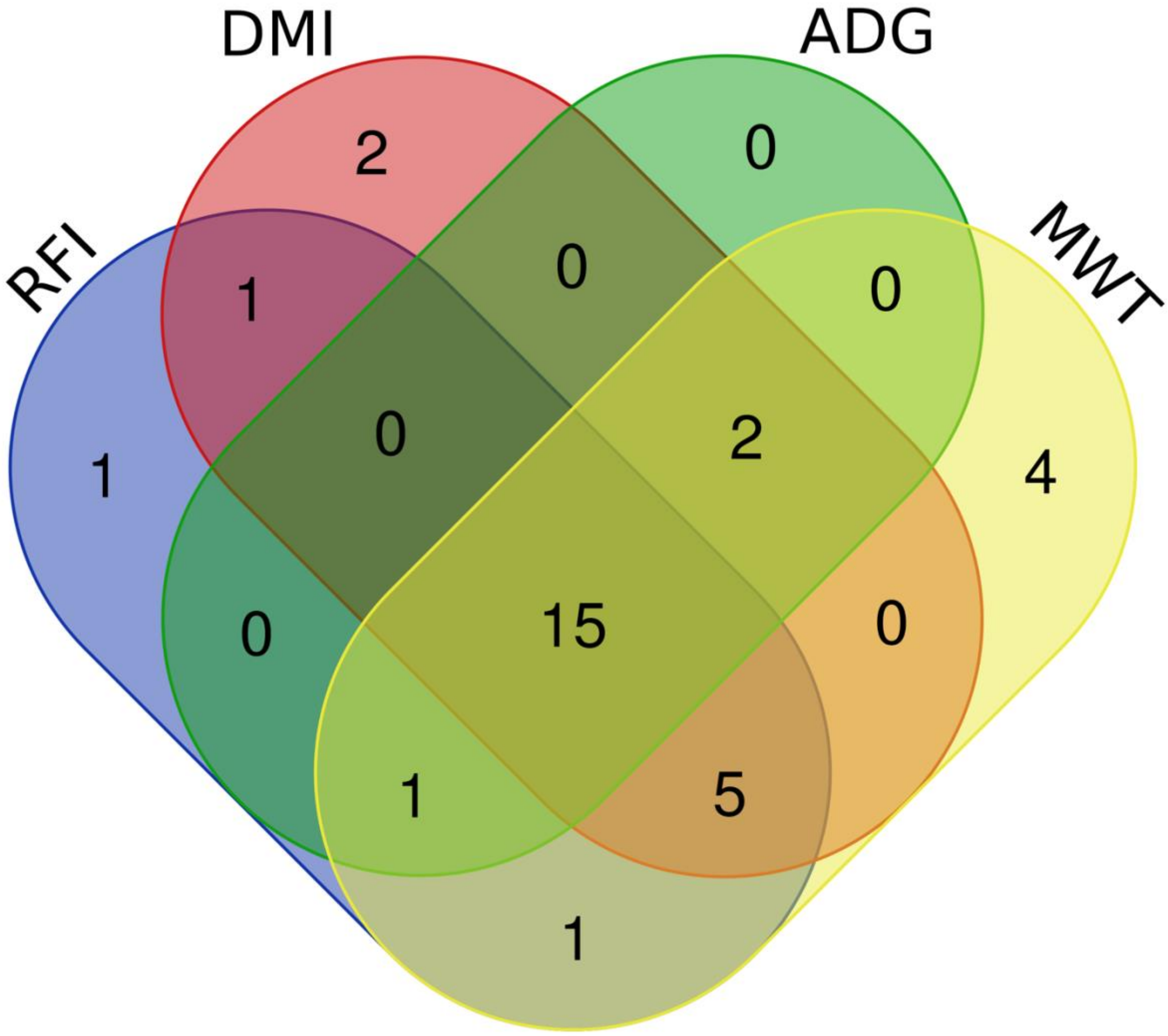

Figure S2 Uniquely common biological functions for feed efficiency traits RFI, DMI, ADG, MWT in a beef cattle multibreed population
